# Cardiotonic Steroids and the Sodium Trade Balance: New Insights into Trade-Off Mechanisms Mediated by the Na^+^/K^+^-ATPase

**DOI:** 10.3390/ijms19092576

**Published:** 2018-08-30

**Authors:** Fatimah K. Khalaf, Prabhatchandra Dube, Amal Mohamed, Jiang Tian, Deepak Malhotra, Steven T. Haller, David J. Kennedy

**Affiliations:** Department of Medicine, University of Toledo College of Medicine and Life Sciences, Health Education Building RM 205, 3000 Arlington Ave, Toledo, OH 43614, USA; kareem.khalaf@rockets.utoledo.edu (F.K.K.); prabhatchandra.dube@utoledo.edu (P.D.); amal.mohamed@rockets.utoledo.edu (A.M.); jiang.tian@utoledo.edu (J.T.); deepak.malhotra@utoledo.edu (D.M.); steven.haller@utoledo.edu (S.T.H.)

**Keywords:** cardiotonic steroids, Na^+^/K^+^-ATPase, inflammation, fibrosis, signaling

## Abstract

In 1972 Neal Bricker presented the “trade-off” hypothesis in which he detailed the role of physiological adaptation processes in mediating some of the pathophysiology associated with declines in renal function. In the late 1990’s Xie and Askari published seminal studies indicating that the Na^+^/K^+^-ATPase (NKA) was not only an ion pump, but also a signal transducer that interacts with several signaling partners. Since this discovery, numerous studies from multiple laboratories have shown that the NKA is a central player in mediating some of these long-term “trade-offs” of the physiological adaptation processes which Bricker originally proposed in the 1970’s. In fact, NKA ligands such as cardiotonic steroids (CTS), have been shown to signal through NKA, and consequently been implicated in mediating both adaptive and maladaptive responses to volume overload such as fibrosis and oxidative stress. In this review we will emphasize the role the NKA plays in this “trade-off” with respect to CTS signaling and its implication in inflammation and fibrosis in target organs including the heart, kidney, and vasculature. As inflammation and fibrosis exhibit key roles in the pathogenesis of a number of clinical disorders such as chronic kidney disease, heart failure, atherosclerosis, obesity, preeclampsia, and aging, this review will also highlight the role of newly discovered NKA signaling partners in mediating some of these conditions.

## 1. Introduction

**Figure 1:**
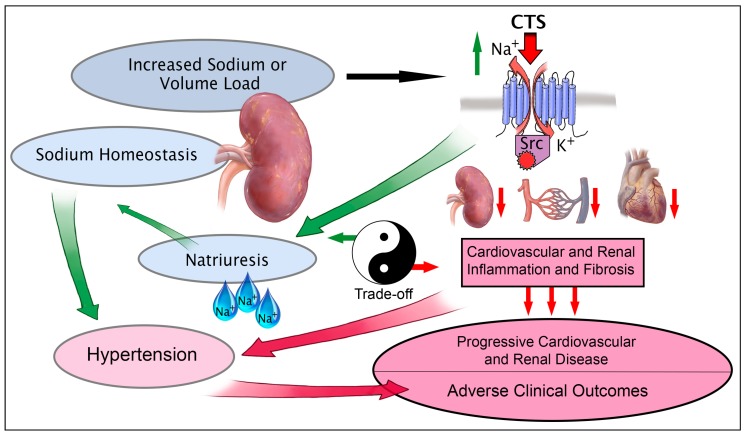
Schematic illustrating the role of the CTS-NKA-Src (cardiotonic steroids- Na^+^/K^+^-ATPase-Src kinase) signaling axis in both its physiologic natriuretic role as well as the trade-off effects induced through stimulation of cardiac, renal, and vascular cell types.

The advent of the discovery of the scaffolding and signaling functions of the NKA (Na^+^/K^+^-ATPase) twenty years ago by Xie and Askari has opened up a multitude of newly appreciated roles for the NKA in both health and disease in almost every major organ system [1,2,3]. Whereas a number of recent reviews have focused on new insights into sodium handling and other physiologically relevant processes directed by NKA signaling [4,5,6,7,8,9], in the current review we will examine the evidence for some of the long-term “trade-offs” of these physiological processes which were originally proposed by Neal Bricker in 1972 [10] (Figure 1). This includes the NKA’s role in inflammation and fibrosis in target organs including the heart, kidney, and vasculature. This review will also highlight the recent developments in what is known about mechanisms of trade-off pathways as they related to CTS-NKA-Src (cardiotonic steroids- Na^+^/K^+^-ATPase-Src kinase) signaling. Recent findings [5,11,12,13], which include the mechanism by which CTS, NKA ligands, can signal through the NKA α-1, have increased the interest in this area significantly. This article will also highlight new developments in what is known about molecular partners of the NKA which help mediate these trade-off pathways. Further, while NKA ligands, such as CTS were first recognized as regulators of renal sodium transport and arterial pressure [14,15], recent findings have highlighted mechanistic links by which CTS modulate interactions of molecular partners with the NKA, especially as this pertains to modulation of immunity, inflammation, and fibrosis [16,17,18]. The objective of the present review is to examine the molecular mechanisms of CTS as they relate to these inflammatory and fibrotic processes.

## 2. Structure and Function of the Na^+^/K^+^-ATPase (NKA)

The cell membrane NKA (or sodium pump) is a member of the P-type family of active cation transport proteins [19]. Initially discovered by the late Jens Skou in 1957 as an ion pump, later studies during the last few decades have shown that the NKA has an essential cell signaling role too [20]. The NKA is the driving power for renal Na^+^ reabsorption and is therefore critically involved in the control of extracellular volume and blood pressure [21,22,23]. The NKA consists of two noncovalently linked polypeptides, the catalytic α subunit (≈110 kDa) and the glycosylated β (≈35 kDa) subunit, and a third recently discovered subunit, the γ (≈10 kDa) subunit, which is a member of the FXYD proteins [24]. The α subunit holds both the ATP and the ligand binding sites, and regulates ATP hydrolysis. As it hydrolyzes ATP, the NKA maintains the ionic gradient via transporting sodium and potassium ions against their concentration gradients. The β subunit is necessary for the assembly of the enzyme, while the α subunit regulates the functionality of the enzyme. Different isoforms of the α and β subunits have been recognized and are believed to have different functions [25]. The α subunit of the NKA has four isoforms (α1, α2, α3, and α4), whereas, three β (β1, β2, and β3) isoforms have been identified. Different groupings of αβ complexes exhibit different functions, characteristics, and sensitivities to different CTS [26]. The α1 isoform can form a signaling complex with signaling proteins such as Src, a tyrosine kinase. This signaling cascade regulates many essential cellular functions, in addition to sodium homeostasis, such as protein trafficking, gene expression and cell growth [27]. This signaling complex can be activated by the binding of CTS [28]. The NKA is the only P-type ATPase that has the ability to bind CTS.

## 3. Cardiotonic Steroids: NKA Ligands Brokering the Sodium Trade Balance

An examination of the role of the NKA in these processes would not be complete without an examination of the ligands for the NKA which drive these signaling events. While these have been reviewed in detail [14,29], here we will briefly summarize some of the most current literature. The digitalis-like factors or endogenous CTS are a class of endogenous volume-sensitive hormones that can be classified structurally into two groups: cardenolides (such as ouabain, digoxin, and digitoxin) and bufadienolides (such as marinobufagenin, telocinobufagin, bufalin, and proscillardin). Cardienolide’s have a five-membered unsaturated lactone/2-pyrone ring at position C17, while bufadienolide’s have a six-membered unsaturated lactone/2-pyrone ring at position C17 [30]. Functionally, it has been shown that the levels and the activity of endogenous CTS in our body vary. Nanomolar concentrations of CTS such as MBG (marinobufagenin) and TCB (telocinobufagin) have been detected in human serum from various volume expanded states [31,32,33,34,35,36,37,38,39,40,41,42,43]. Ouabain (or ouabain-like material which is immune-reactive with ouabain antibodies) has been detected in the serum of patients with hypertension [41,44], congestive heart failure [45], and terminal renal failure [46]. While both marinobufagenin and telecinobufagin were detected in the serum of patients with end stage renal failure [43], telocinobufagin (TCB) was found at a higher concentration than that of marinobufagenin. Other studies have found that TCB has the most potent activity when compared to other CTS, and has the greatest suppressive effect on NKA [47,48,49]. In Table 1, we list CTS levels in blood, urine, and tissue samples across species and various pathologies as summarized from several key references above. Importantly these levels should be interpreted in light of the known differences in the affinity of CTS for the NKA across species.

CTS have a natriuretic effect and they have been known to regulate renal homeostasis. Therefore, endogenous CTS levels increase in response to volume expansion states accompanying chronic diseases, such as hypertension, heart failure, and renal disease. However, unintended effects of high concentrations of these hormones potentially contribute to disease progression [10,29,50,51,52]. Long-lasting elevation of CTS may produce “off-target” effects [13], potentially including the profibrotic and the proinflammatory effects of these hormones [53]. Hence, chronic elevation of CTS signaling through NKA has implications not only for natriuretic response to high salt and volume load, but also for pathological adaptation to these conditions [54]. Clinical and experimental evidence from our group and others has also demonstrated the profibrotic effects of these hormones in both cardiac and renal tissue [39,55,56]. When considered together, we argue that these studies strongly implicate CTS role in inflammation and fibrosis associated with chronic conditions. The aim of the current review is to evaluate the proinflammatory and the profibrotic effects of CTS and the mechanism by which it does so, as detailed by different investigators.

## 4. CTS (Cardiotonic Steroids), NKA, and Fibrosis

**Figure 2:**
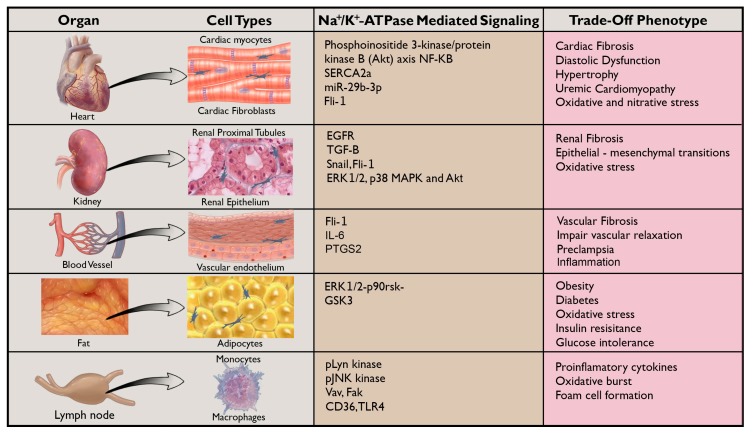
Summary of the known proinflammatory and profibrotic signaling mechanisms mediated by the CTS-NKA-Src signaling axis in cardiac [44,63,73,74,75,76,77,78,80,81,82], renal [32,39,43,51,52,55,63,64,65]], vascular [69,70,92], adipocyte [93,94,95,96,97,98,99,100,101,102,103,104,105], and immune cells [16,56].

Fibrosis in its own right can be conceptualized as a trade-off imbalance between extracellular matrix production and degradation that occurs in almost all types of chronic disease [57,58]. Several pathways and mechanisms have been described in the pathogenesis of fibrosis [58,59,60,61,62]. Recent studies have examined the role of the NKA signaling in mediating organ fibrosis. Here we will briefly review some of the most current literature that highlights the role of CTS with respect to NKA signaling in mediating fibrosis in target organs including the kidney, heart, and vasculature (Figure 2). In 2009, Fedorova and coworkers showed that MBG administration induces renal fibrosis in rats. MBG targets different populations of renal cells, which then activates interstitial fibroblasts and increases collagen expression [32]. The study also showed that MBG upregulates Snail, a transcription factor that has been implicated in the differentiation of epithelial cells into mesenchymal cells (epithelial–mesenchymal transition or EMT) [51,52]. This further subsidizes the expansion of fibrosis. Elkareh and colleagues further demonstrated that nanomolar concentrations of MBG stimulate collagen synthesis and induce fibrosis in kidney and cardiovascular tissues through the NKA–Src–EGFR (Na^+^/K^+^-ATPase- Src kinase-Epidermal growth factor receptor) signaling cascade [39,63]. In animal models, we have shown that infusion of CTS to a level similar to that seen in rodents with a partial nephrectomy activates the NKA-Src-EGFR, ERK (extracellular-signal-regulated kinase) and many other biochemical and physiological features similar to those seen in patients with uremic cardiomyopathy. Interestingly, they revealed that active immunization against CTS attenuates most of the uremic cardiomyopathy features. Additionally, Haller in 2013 showed that passive immunization against MBG significantly improved renal function and markedly reduced renal fibrosis following experimental induction of renal disease [55]. This further highlighted CTS role in the pathophysiology of CKD (chronic kidney disease) and paved the road for a new therapeutic target. Immunization against CTS might serve as a potential treatment in these high risk populations. Cheng and coworkers showed that targeting the NKA-mediated signaling could attenuate renal fibrosis. He demonstrated that suppression of Src activation and its downstream ERK1/2, p38 MAPK (mitogen activated protein kinase) and Akt (protein kinase B) signaling pathways can effectively attenuate UUO (unilateral ureteral obstruction)-induced renal fibrogenesis [64]. This profibrotic pathway of MBG also involves activation of Fli-1, a nuclear transcription factor that negatively regulates collagen synthesis [65]. In addition to this, studies that have linked the association of elevated CTS levels to renal disease and decline in renal function provide many other arguments in favor of CTS role in renal disease. Komiyama and coworkers showed that patients with end stage renal disease (ESRD) have high level of CTS in their plasma as confirmed by LC-MS [43]. Kennedy and colleagues showed that animals subjected to partial nephrectomy demonstrated elevation in CTS levels similar to that seen in patients with renal failure [39]. Adding to that, other studies have highlighted the role of CTS in different renal related conditions such as hypertension. Increased levels of CTS have also been demonstrated in pregnancy and implicated in the pathogenesis of pregnancy-induced hypertension [66,67,68,69]. CTS binding to the receptor site on the α-subunit of the NKA induces EGFR-dependent cellular signaling, which is involved in the CTS mediated pathology. Further neutralizing the effects of endogenous CTS, using intravenously administered Digibind, an anti-digoxin antibody, improves renal function by reducing the NKA inhibitory activity of CTS in plasma in patients with severe preeclampsia [66,67,68,69,70]. 

In addition to CTS role in mediating renal fibrosis, a number of studies, in vivo and in vitro, have shown that CTS have the capacity to induce signaling cascades, which are directly involved in the development of fibrosis in other organs, such as heart, vessel, and skin. In 2007, Elkareh showed that MBG and other cardiotonic steroids, such as ouabain and digoxin, promote collagen synthesis in cardiac fibroblasts, and induce an increase in procollagen-1 mRNA expression along with an increase in collagen synthesis [63]. They revealed that, in normal animals, infusing CTS in a concentration similar to the circulating levels seen in patients with renal failure stimulates collagen synthesis in cardiac fibroblast primary culture, and that active immunization against MBG significantly attenuated the development of cardiac fibrosis in vivo. They also showed that the induction of collagen production by CTS depended on the integrity of signaling through the NKA-Src cascade, as disruption of Src kinase signaling via administration of Src kinase inhibitors diminished the CTS induced profibrotic effects. Additionally, the blockade of NKA signaling with immunization as well as pharmacologic inhibitors effectively reduced the oxidative stress seen with experimental renal failure. Grigorova and Fedorova further showed that rats on a high salt diet developed aortic fibrosis [71,72]. These studies showed that this phenotype was mediated through MBG-dependent mechanisms, and was reduced by immune-neutralization of MBG.

Recent work by our group showed that in experimental cardiomyopathy, cardiac fibrosis is also related to increase levels of circulating MBG [73]. Given that MBG signals through the NKA and NKA signaling is known to stimulate the mTOR system, which has been implicated in the development and progression of renal disease, we speculated that Rapamycin, an inhibitor of mTOR, may significantly attenuate the cardiomyopathy induced by partial nephrectomy or MBG infusion. In fact, the results showed that rapamycin treatment in these settings significantly attenuated profibrotic signaling and cardiac fibrosis [73]. Furthermore, we have demonstrated that the regulation of miR-29b-3p through NKA signaling is in part necessary for the development of CTS induced cardiac fibrosis [74]. Drummond and coworkers showed, for the first time, that signaling through the NKA regulates miR-29b-3p expression both in vivo and in vitro. Along with this, CTS can also trigger the phosphoinositide 3-kinase/protein kinase B (Akt) axis, stimulate NF-κB, and increase the intracellular Ca^2+^ concentration all of which have established links to organ fibrosis as well [3].

Furthermore, many studies [75,76,77,78] showed that in rats, chronic peripheral administration of low doses of ouabain induced cardiac hypertrophy and fibrosis. Meanwhile, passive immunization against CTS significantly ameliorates the progression of cardiac hypertrophy and fibrosis [79]. Several studies have shown an association between CTS levels and cardiac geometry and that advanced stages of hypertrophy were associated with elevated plasma CTS [78,80,81]. Pierdomenico and colleagues found that plasma CTS levels were significantly higher in patients with LV (left ventricular) hypertrophy compared to patients with normal LV geometry [44]. Interestingly, passive immunization against CTS, in animal models with myocardial infraction, significantly attenuates the progression of LV dilation and improve LV function [82]. Additional work in this same area also showed that ouabain infusion induced LV hypertrophy [78]. 

Importantly, while the current review has focused primarily on the non-ion dependent signaling mechanisms of the NKA, the [Na^+^]i/[K^+^]i ratio has been shown to also initiate similar signaling events. A number of important studies have shown the role of NKA inhibition and elevation of the [Na^+^]i/[K^+^]i ratio in the activation of Src-EGFR-MAPK- and Akt-mediated signaling pathways triggered by CTS, and readers are referred to a recent review which more thoroughly addresses this topic [83]. The importance of this concept is highlighted by recent data showing that NKA inhibition by CTS triggers TGFβ-induced fibrosis in cultured human lung fibroblasts via [Na^+^]i/[K^+^]i-mediated signaling which results in augmented expression of COX-2 [84] and downregulation of TGFβ2R [85]. Cumulatively, these studies have broadened the understanding of the role of CTS signaling through the NKA in human disease. Many follow up studies have linked the association of CTS signaling to other health issues, such as preeclampsia. In 2010, Fedorova showed that plasma levels of CTS in patients with preeclampsia are elevated on average, four-times that of normal patients [69]. Later studies done by Nikitina and coworkers revealed that in preeclampsia, elevated levels of endogenous MBG induce vascular fibrosis and impairment of vascular relaxation through a Fli-1-dependent mechanism [70]. The study showed that MBG induced vascular fibrosis in umbilical arteries similar to that seen in preeclampsia. Uddin and colleagues have described several studies not only confirming elevations of CTS in preeclampsia [86] but also detailing the proapoptotic and antiproliferative effects of CTS such as MBG in the disruption of normal placental function [87,88,89,90]. Recently, Lenaerts and coworkers have developed an LC-MS method to detect and definitively identify MBG in plasma samples and confirmed the presence of endogenous MBG in preeclampsia [91]. This is significant, because it was a highly specific assay, which positively identified MBG using multiple MRM (multiple reaction monitoring) transitions, providing very powerful and direct evidence of the presence of this CTS in a physiologically relevant human setting. The utility of LC-MS/MS will allow researchers to study the role of MBG and other CTS in mediating trade-off effects associated with other volume expanded states in which these hormones have been implicated (Figure 2). 

## 5. CTS, NKA, and Inflammation

Inflammation and oxidant stress play a dominant role in the onset and progression of organ injury in chronic conditions [106]. Inflammation induces the release of cytokines and enhances the expression of adhesion molecules, which together contribute to recruitment of more inflammatory cells that ultimately aggravate the condition extensively. Current studies have examined the role of the CTS signaling through NKA in mediating inflammation and oxidative stress in chronic conditions (Figure 2). Here we will highlight the role of CTS with respect to NKA signaling in oxidative stress and immune modulation. Over the last five years, many studies have suggested a relationship between CTS and inflammation. There is some evidence that circulating levels of CTS are elevated during the inflammatory response [16] and many studies have linked specific cardiac glycosides to a proinflammatory response. Others have shown evidence of CTS inducing an anti-inflammatory response. 

It is well established that CTS signal through the NKA and are important in the regulation of renal sodium transport and arterial pressure [107,108,109,110]. However, recent work implicates CTS in the modulation of immunity, more specifically as mediators of inflammation. Ouabain is a well-characterized CTS and its role as a NKA inhibitor is associated with its cardiovascular effects [107,108,109,110]. Ouabain has also recently been shown to induce inflammation as a result of this inhibitory property. Goncalves-de-Albuquerque and colleagues demonstrated that intratracheal administration of ouabain induces lung inflammation in mice via inhibition of the NKA in alveolar cells [111]. Ouabain and other CTS have been associated with cell signaling mechanisms in immune cells as well. Quastel and Kaplan first identified a relationship between ouabain and the immune system when they demonstrated that ouabain inhibits lymphocyte proliferation induced by the mitogen phytohaemagglutinin [112]. This was clearly repeated using different stimuli and thus confirmed [18,113,114,115]. The studies mentioned thus far have demonstrated a relationship between CTS and inflammation [12], however, investigators are currently looking into the specific mechanisms by which chronic elevation of CTS levels induce an inflammatory response. Recent data has been collected on the effect of CTS on cells that respond to chronic inflammation such as macrophages, mast cells, and monocytes [116]. Our group hypothesized that CTS enhance interactions between immune cells and endo/epithelial cells through the NKA and Src kinase signaling pathway. After examining the effect of CTS on the expression of the biological markers associated with adhesion in both immune and endo/epithelial cells, we found that the CTS telocinobufagin (TCB) enhanced the expression of the β2 integrin family members CD11b/CD18 and induced the expression of intercellular adhesion molecules I-CAM (intercellular adhesion molecule) and V-CAM (vascular adhesion molecule). Furthermore, when testing for macrophage adhesion on two stable cell lines that contained either NKA α-1 (wild type) or 90% NKA α-1 knockdown, we found that the TCB induced macrophage adhesion was diminished >80% in NKA α-1 knockdown cells. Thus suggesting that CTS potentiates immune cell activation and adhesion to the endo/epithelium through an NKA-α-1-Src dependent mechanism [12]. The CTS bufalin and ouabain have been shown to induce apoptosis in human leukemia cells, although bufalin has demonstrated a more potent effect [117,118]. It has been suggested that the extracellular signaling regulated kinases (ERK)-kinase cascade is excessively activated in order for bufalin-mediated apoptosis to occur [112,119,120]. Kurosawa and colleagues found that treating human leukemia THP-1 cells with bufalin induced inflammatory cytokines interleukin-1β (IL-1β) and tumor necrosis factor-α (TNF-α). After treating the cells with an inhibitor of ERK, PD-98059, they found that the cytokine production was attenuated, suggesting that the ERK pathway is responsible for the inflammatory response induced by bufalin [119]. Bufalin’s ability to induce cytokine production suggests that CTS is capable of participating in an inflammatory response. On the other hand, Zhakeer and colleagues suggested that bufalin exhibits anti-inflammatory effects. Their group developed a mouse asthma model and determined cytokine recruitment using an enzyme-linked immunosorbent assay. They found that mice treated with bufalin showed a significant decrease in total inflammatory cells as well as IL-4, IL-5, and IL-13 [121]. Thus, more work is necessary to resolve these apparent discrepancies and to further refine if and how CTS mediate the inflammatory response in these settings. Along these lines, readers are referred to a recent review of the evidence for both pro- and anti-inflammatory activities of the CTS ouabain [18].

The transcription factor, nuclear factor kappa-light-chain-enhancer, of activated B cells (NF-κB) is considered a master regulator of immunity [16]. The NF-κB pathway is activated in macrophages when they recognize a pathogen, inducing a multitude of proinflammatory responses. Although the mechanisms related to NF-κB activation are not completely understood, activation of this pathway is associated with a proinflammatory response. Using human monocyte-derived macrophages (HMDM), as well as murine peritoneal macrophages, Chen and colleagues demonstrated that the CTS ouabain activates the NF-κB pathway leading to proinflammatory cytokine production [16]. They found that 25 nmol/L ouabain increased NF-κB-transcriptional activity up to three-fold. They then used a quantitative real time-polymerase chain reaction to show that ouabain increased expression of monocyte chemotactic protein 1 (MCP-1), TNF-α, prostaglandin endoperoxide synthase 2 (PTGS2), chemokine (CC motif) ligand (CCL5), IL-6, IL-1β, ICAM1, CXCL10, and CXCL9 [16]. Interestingly, five years prior to this publication, the sharp elevation of PTGS2 and IL6 expression triggered by ouabain was found in rat vascular smooth muscle cells, human endothelial cells, and the HeLa cell line [92]. Importantly, in these cells augmented expression of PTGS2 and IL6 was mediated by NKA inhibition and elevation of the [Na^+^]i/[K^+^]i ratio. In addition to these findings, the neurotoxicity of Venenum Bufonis has been linked to neural inflammation caused by activation of the NF-κB transcription factor [122]. However, the literature seems to be divided on this topic. Although these studies show CTS activating the pathway, others show CTS actually having an inhibitory effect on the transcription factor. For example, Wang and colleagues found that digoxin inhibited NF-κB and that TNF-α-stimulated NF-κB activity and suppressed NF-κB initiating genes (Bcl-2, Bcl-xL, cyclin D1, and c-myc) [123]. It is important to note that this study was done on Burkitt’s lymphoma cells in order to study the potential for digoxin as a therapeutic agent for cancer, while the other studies mentioned were looking at the specific immune cell response associated with elevated levels of CTS. Importantly, there are key differences between digoxin and CTS such as ouabain including the apparent inability of digoxin to activate Src kinase [124]. Additionally, according to many epidemiological studies, there is a growing relationship between digoxin treatment and increased mortality and a suggestion that volume expanded settings in which CTS are elevated may contribute to digoxin toxicity [49,125]. Some investigators believe that this link is due to the proinflammatory nature of CTS in patients with cardiac and renal disease. Kobayashi and colleagues found that ouabain induced cardiac inflammatory responses, such as macrophage infiltration and IL-1β release when mice were primed with LPS (lipopolysaccharide) [126]. They ultimately found deficiency of NLRP3 and caspase-1 attenuated ouabain dysfunction and inflammation. Given these intriguing findings, the pro-and anti-inflammatory effect of CTS on cardiac and renal disease, especially in volume expanded conditions where elevated levels of CTS persist, is a topic that warrants further investigation. 

## 6. New Horizons for NKA Signaling: Aging, Obesity, Diabetes, and Atherosclerosis

The amplification of oxidant stress by NKA has emerged as a key theme in the role that the NKA plays in the pathophysiologic trade-off adaptations to volume expansion [5,54,101,127]. Oxidative stress has an essential role in the pathogenesis of many clinical disorders such as obesity, atherosclerosis, diabetes, and aging. Accordingly, we will briefly summarize some of the most current literature that highlights the role of NKA signaling in the pathogenesis and progression of these conditions. 

Obesity is a global epidemic, and can be defined as an abnormal or excessive accumulation of fat that impairs health. Obesity is the leading cause of morbidity and mortality associated with cardiovascular and metabolic syndrome [128,129]. Numerous studies have shown that systemic oxidative stress is a main element that generates and maintains the pathological consequences of obesity both in vitro and in vivo [93,95,96,130]. Because the NKA can amplify oxidant signaling, scientists speculate that the NKA signaling plays a role in oxidative stress related to obesity. In fact, studies have shown that a peptide designed to inhibit NKA signaling can ameliorate obesity. One of the most important developments in this field was the development of a specific peptide inhibitor of the NKA-Src signaling axis, termed pNaktide [97]. Using this specific inhibitor, Sodhi and coworkers showed that in 3T3-L1 preadipocytes, pNaKtide attenuated oxidant stress and lipid accumulation in a dose-dependent manner [101]. Additionally, they found that, in mice fed a high-fat diet, administration of pNaKtide reduced body weight, restored systemic redox and inflammatory milieu, and improved insulin sensitivity. This study highlighted the role of the NKA signaling cascade to amplify reactive oxygen species involved in adipogenesis, a process not previously linked to the NKA signaling. Earlier studies by Turaihi and colleagues showed that in obesity, the quantity of NKA sites on leucocyte membranes are significantly increased, and that this is associated with accelerated 86Rb transport [103]. Interestingly, both of these indices decreased following 4% to 5% reduction in body weight; however, this group did not specifically link the increase in NKA quantity as central component to the pathogenesis of obesity. However, a more recent study demonstrated that the NKA/ROS (reactive oxygen species) amplification loop contributes significantly to the development and progression of obesity and that visceral adipocytes create systemic oxidant stress through the feed-forward oxidant amplification loop of the NKA-Src-EGFR signaling [101]. In fact, Iannello and coworkers showed that blockade of this amplification with pNaKtide ameliorates oxidative stress and obesity in mice subjected to a high-fat diet [131]. Another study by Martin and colleagues showed that adipogenic markers PPARγ, FAS, and C/EBP in the visceral fat of western-diet fed mice was significantly reduced after lentiviral-mediated adipocyte-specific delivery of pNaKtide, which inhibits NKA signaling [98]. 

The NKA/ROS amplification loop is implicated in the pathogenesis of other conditions such as atherosclerosis and diabetes in addition to its role in obesity. Atherosclerosis is a worldwide epidemic and leading cause of death in developed countries. Atherosclerosis is marked by inflammation and the formation of plaque within arterial walls. Given the importance of oxidative stress in the pathophysiology of atherosclerosis, and the known ability of the NKA to act as an amplifier for ROS, several studies have investigated the role of NKA signaling in this setting. Indeed, one study demonstrated that administration of pNaKtide in ApoE^−/−^ mice fed a western diet significantly decreased plasma ALT (alanine aminotransferase), triglycerides, FFA (free fatty acide), and LDL (low density lipoprotein) levels [102]. Further, the study showed that ApoE^−/−^ mice fed a western diet had decreased plasma HDL (high density lipoprotein) levels, and this decrease was reversed by pNaKtide. ROS levels and plaque size were significantly reduced by pNaKtide treatment as well. Furthermore, adipocyte dysfunction in mice fed a western diet was also prevented by lentiviral-mediated adipocyte-specific delivery of pNaKtide [98]. The results showed that Lenti-adipo-pNaKtide significantly reduced western diet-induced weight gain, along with visceral and subcutaneous fat content. Additionally, the increase in cardiac hypertrophy in high fat fed animals was attenuated with lenti-adipo-pNaKtide. On this background, Sodhi and colleagues showed that the administration of pNaKtide to mice fed a western diet containing high amounts of fat and fructose significantly reduced obesity as well as hepatic steatosis, inflammation, and fibrosis [102]. The study also revealed an improvement in mitochondrial fatty acid oxidation, insulin sensitivity, dyslipidemia, and aortic streaking in their model. To further examine the role of the NKA in the development of atherosclerosis, similar studies were performed in ApoE knockout mice exposed to a western diet. In these mice, pNaKtide not only significantly improved atherosclerosis, but also ameliorated steatohepatitis, dyslipidemia, and insulin sensitivity. These studies complement the fundamental observation which we will review in the next section that the NKA participates in atherogenesis [132].

Diabetes mellitus is another inflammation-related process that appears to be increasing rapidly, threatening to reduce life expectancy for humans worldwide [99,105]. Inflammation and oxidative stress have been implicated in both development and progression of diabetes [94,99,104]; although not much is known about the role of the NKA in this setting. However, studies have shown that inhibition of NKA signaling using pNaKtide improved glucose tolerance, insulin sensitivity, and HOMA-IR scores in ApoE^−/−^ mice fed a western diet [102]. A different study showed that glucose tolerance improved in mice fed a western diet after lentiviral-mediated adipocyte-specific delivery of pNaKtide [98]. This study suggests that the NKA/ROS amplification loop may be involved in development of the diabetes phenotype as well. 

The NKA oxidant amplification loop may be involved in advancing the aging process in both in vivo and in vitro models as well. In fact, mice fed a western diet showed oxidant injury and aggravated functional and morphological aging markers, whereas treating with pNaKtide reduced these changes [54]. Collectively, these studies strongly suggest that the NKA/ROS amplification loop contributes significantly to the development and progression of inflammation and oxidative stress related to obesity, atherosclerosis, diabetes, and aging (Figure 2). More studies are needed to unravel the development of new therapeutic targets for these conditions. 

## 7. Novel Signaling Partners of the NKA in Inflammation and Fibrosis

The NKA has an important role in cell signaling through its interactions with endogenous CTS and signaling molecules such as Src kinase [14,133]. CTS and other ligands of this receptor complex are known to be involved in initiation and magnification of signaling cascades through the recruitment and assembly of a cell-specific NKA signalosome [134]. In the last few decades, many studies have revealed the role of the NKA and its signaling complexes in various diseases including atherosclerosis, inflammation, and fibrosis. Research efforts are being made to study different signaling partners of the pump and to unravel molecular mechanisms involved in the pathogenesis of various diseases. Our group has shown that hyperlipidemia and obesity triggers an inflammatory paracrine loop between proximal tubule cells and their associated macrophages, which is dependent on scavenger receptor CD36 and the NKA [56]. CD36 is a key scavenger receptor which is expressed on variety of cell types including monocytes, macrophages and proximal tubule cells and mediates inflammation in pro-atherogenic conditions [135,136]. CD36 also plays an important role in uptake of the pro-atherogenic lipoprotein and oxidized LDL, which is elevated in hyperlipidemic conditions like uremia [137,138]. We have shown that CD36 and the NKA α-1 colocalize in both renal proximal tubule cells and macrophages. Further, we have demonstrated that ligands generated during hyperlipidemic states (such as oxidized LDL and CTS) can activate CD36 and the NKA α-1 that triggers an inflammatory signaling loop between renal proximal tubule cells and their associated macrophages. This leads to amplification of chronic inflammation, oxidant stress, and fibrosis causing renal dysfunction, a common sequellae of pro-atherogenic and hyperlipidemic states. 

Chen and colleagues further showed that the NKA plays a significant role in oxidized LDL-CD36 signaling axis, specifically in macrophages. They confirmed the NKA as a key binding partner of CD36 in this cell type and demonstrated that oxLDL binding to CD36 recruited and activated Lyn kinase through the NKA. Here, activation of NKA associated Lyn kinase leads to foam cell formation and contributes to the development of atherosclerosis by inhibiting macrophage migration and trapping macrophages in the neointima. Macrophages deficient in the α1 subunit of the NKA resulted in a significant decrease in oxLDL uptake, foam cell formation, and oxLDL-induced inhibition of cell migration, suggesting that it is a target for anti-atherosclerotic lesion development. Chen [132] further showed that CTS trigger an inflammatory response in murine and human macrophages by activating NF-κB, causing proinflammatory cytokine production in these primary macrophages through a signaling complex, including CD36, NKA, and Toll-like receptor 4 (TLR4). TLR4 is also involved in recruiting MyD88, an adaptor protein for NF-κB activation. Their data showed that macrophages deficient in NKA, scavenger receptor CD36, or TLR4 were resistant to CTS-induced NF-κB activation, indicating the crucial role of these three receptors in the proinflammatory pathway. 

Our group has also reported a novel signaling mechanism involving CD40 regulation by NKA [24]. CD40 is a membrane glycoprotein and member of tumor necrosis factor receptor superfamily and expressed on a variety of cells including B-lymphocytes, macrophages and monocytes, dendritic cells, and endothelial cells [139]. The soluble form of the CD40 ligand expressed and secreted by activated platelets primarily activates CD40 and is elevated in atherosclerosis and renal injury [140,141]. CD40 receptor activation on the proximal tubular epithelium of the kidney contributes to fibrosis and inflammation in various models of kidney injury [142]. Xie and colleagues showed that knockdown of the α1 subunit of NKA leads to reduced expression of CD40, while rescue of the α1 subunit restores CD40 expression in renal epithelial cells [24]. Disruption of the NKA-Src complex also interrupts CD40 signaling. Given the role of the NKA-Src complex in the pathogenesis of renal injury and fibrosis, these findings suggest that the NKA and CD40 may be a part of profibrotic signaling in the kidney and inhibition of this pathway may be useful in the treatment of renal fibrosis. 

**Figure 3:**
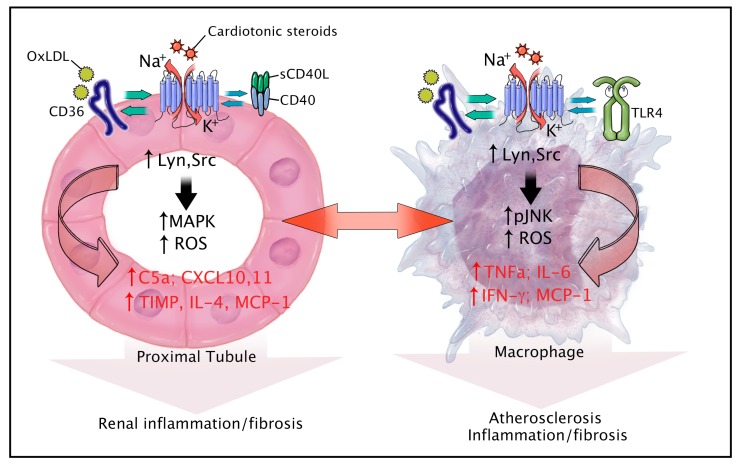
Summary of novel signaling interactions identified between the NKA and other cell surface receptors in renal epithelial and immune cell types [14,24,56,132,133,134,135,136,137,138,139,140,141,142,143].

A common theme emerges for both CD36 and CD40 studies in that both of these receptors (a) have little intracellular presence by which to direct signaling events, (b) lack intrinsic kinase or phosphatase activity, (c) have no known intracellular scaffolding domain(s), (d) no direct connection with GTPases, (e) reside (at least in part) in caveolae, and (f) rely on activation of Src family kinases to mediate signaling. Thus the ability of both of these receptors to interact with the signaling NKA may prove to be a common denominator which allows these receptors to activate multiple signaling pathways. If this is true, there are a host of implications not only for the signaling events and biology of these two receptors, but for a host of similarly configured receptors throughout the body. The propensity of the NKA to interact with and facilitate the signaling of multiple proteins involved in atherogenesis, inflammation, and fibrosis makes it a valuable target for therapy in different diseases. A summary of novel signaling interactions identified between the NKA and other cell surface receptors in renal epithelial and immune cell types is presented in Figure 3.

## 8. Summary

Xie’s discovery of the scaffolding and signaling functions of the NKA twenty years ago has uncovered new and unexpected roles not only in directing sodium handling but also in some of the long-term “trade-offs” of these physiological processes. The involvement of the NKA as a key player in end organ inflammation and fibrosis in volume expanded conditions make this discovery clinically important. Further, appreciation of the molecular partners which interact with the NKA and help mediate these pathways suggest unique therapeutic targets for modulating these trade-off effects. 

## Figures and Tables

**Table 1:** Summary of cardiotonic steroid levels as measured in various biological matrices.

Cardiotonic Steroid	Concentration	Biological Matrix	Condition	Species	References
MBG	12.3 ± 1.7 nmol	Urine	Acute myocardial ischemia	Human	[31]
MBG	4.2 ± 0.8 nmol	Urine	Angina pectoris	Human	[31]
MBG	1.9 ± 0.38 nmol/L	Plasma	Acute myocardial ischemia	Human	[31]
MBG	0.51 ± 0.07 nmol/L	Plasma	Angina pectoris	Human	[31]
MBG	0.38 ± 0.1 nmol/L	Plasma	Healthy	Human	[31]
MBG	0.49 ± 0.05 nmol/L	Plasma	Volume expansion	Rat	[33]
MBG	0.20 ± 0.06 nmol/L	Plasma	Healthy	Rat	[33]
Ouabain	0.0032 ± 0.0023 nmol/g	Pituitary	Healthy	Rat	[33]
Ouabain	0.0309 ± 0.00312 nmol/g	Pituitary	Volume expansion	Rat	[33]
Ouabain	0.21 ± 0.04 nmol/L	Plasma	Healthy	Rat	[33]
Ouabain	0.09 ± 0.02 nmol/L	Plasma	Volume expansion	Rat	[33]
MBG	0.00007 ± 0.00002 nmol/g	Pituitary	Healthy	Rat	[33]
MBG	0.00005 ± 0.00001 nmol/g	Pituitary	Volume expansion	Rat	[33]
Ouabain	0.138 ± 0.043 nmol/L	Plasma	Healthy	Human	[36]
Ouabain	0.037 ± 0.007 nmol/L	Plasma	Healthy	Dog	[36]
Ouabain	0.0386 nmol/g	Adrenal	Healthy	Rat	[36]
Ouabain	0.0051 nmol/g	Pituitary	Healthy	Rat	[36]
Ouabain	0.0025 nmol/g	Hypothalamus	Healthy	Rat	[36]
Ouabain	0.0025 nmol/g	Atria	Healthy	Rat	[36]
Ouabain	0.0034 nmol/g	Kidney	Healthy	Rat	[36]
Ouabain	0.0021 nmol/g	Liver	Healthy	Rat	[36]
Ouabain	0.08 ± 0.018 nmol/L	Plasma	Healthy	Rat	[36]
Ouabain	0.04 ± 0.012 nmol/L	Plasma	Adrenalectomy	Rat	[36]
Ouabain	0.12 ± 0.043 nmol/L	Plasma	Uninephrectomy + salt	Rat	[36]
Ouabain	0.98 ± 0.079 nmol/L	Plasma	DOCA + salt	Rat	[36]
Ouabain	0.20 ± 0.062 nmol/L	Plasma	5/6^th^ Nephrectomy	Rat	[38]
Ouabain	0.12 ± 0.062 nmol/L	Plasma	Healthy	Rat	[38]
MBG	0.36 ± 0.016 nmol/L	Plasma	Healthy	Rat	[39]
MBG	0.57 ± 0.036 nmol/L	Plasma	5/6^th^ Nephrectomy	Rat	[39]
MBG	0.03 ± 0.0023 nmol	Urine	Healthy	Rat	[39]
MBG	0.06 ± 0.0045 nmol	Urine	5/6^th^ Nephrectomy	Rat	[39]
Ouabain	0.43 ± 0.053 nmol/L	Plasma	Healthy	Rat	[39]
Ouabain	0.44 ± 0.043 nmol/L	Plasma	5/6^th^ Nephrectomy	Rat	[39]
Ouabain	0.012 ± 0.0015 nmol	Urine	Healthy	Rat	[39]
Ouabain	0.011 ± 0.0019 nmol	Urine	5/6^th^ Nephrectomy	Rat	[39]
Ouabain	0.25 ± 0.053 nmol/L	Plasma	Healthy	Human	[42]
Ouabain	0.38 ± 0.019 nmol/L	Plasma	Essential Hypertension	Human	[42]
Ouabain	0.04 ± 0.0002 nmol/L	Plasma	Heart Failure	Human	[45]
Ouabain	1.59 ± 2.2 nmol/L	Plasma	Heart Failure	Human	[45]
MBG	2.3 ± 0.7 nmol/L *	Plasma	Healthy	Human	[47]
MBG	9.5 ± 4.8 nmol/L *	Plasma	End Stage Renal Disease	Human	[47]
TCB	4.4 ± 1.4 nmol/L *	Plasma	Healthy	Human	[47]
TCB	17.0 ± 10.7 nmol/L *	Plasma	End Stage Renal Disease	Human	[47]

MBG, Marinobufagenin; TCB, Telecinobufagenin; DOCA, deoxycorticosterone acetate treated uninephrectomized rat. * Denotes measures obtained by quantitative LC-MS-MS.

## Abbreviations

BP	Blood pressure
CKD	Chronic kidney disease
CTGF	Connective tissue growth factor
CTS	Cardiotonic steroid
HDL	High density lipoprotein
HF	Heart Failure
MBG	Marinobufagenin
NKA	Na^+^/K^+^-ATPase
PNx	Partial (5/6th) Nephrectomy
ROS	Reactive oxygen species
Src	Sarcoma viral oncogene kinase
TCB	Telocinobufagin
UUO	Unilateral ureteral obstruction
